# Cardiac left ventricular myocardial tissue density, evaluated by computed tomography and autopsy

**DOI:** 10.1186/s12880-019-0326-4

**Published:** 2019-04-12

**Authors:** Alexandra G. Gheorghe, Andreas Fuchs, Christina Jacobsen, Klaus F. Kofoed, Rasmus Møgelvang, Niels Lynnerup, Jytte Banner

**Affiliations:** 1 Section of Forensic Pathology, Department of Forensic Medicine, University of Copenhagen, Frederik V’s vej 11, 1 sal, 2100 Copenhagen, Denmark; 2 Department of Cardiology, The Heart Centre, Rigshospitalet, University of Copenhagen, Copenhagen, Denmark; 3 Department of Radiology, Diagnostic Centre, Rigshospitalet, University of Copenhagen, Copenhagen, Denmark

**Keywords:** Left Ventricular Shell Volume, Left Ventricular Mass, Anatomic Left Ventricular Mass, Computed Tomography, Left Ventricular Mass, Myocardial Tissue Density, Post Mortem Computed Tomography

## Abstract

**Background:**

Left ventricular mass (LVM) is an independent risk factor for the prediction of cardiac events. Its assessment is a clinically important diagnostic procedure in cardiology and may be performed by Computed Tomography (CT). The aim of this study was to assess the correlation between the cardiac left ventricular shell volume (LVShV) determined by postmortem Computed Tomography (PMCT) and the anatomic LVM obtained at autopsy and to calculate the myocardial tissue density.

**Methods:**

A total of 109 deceased individuals were examined with a 64-slice CT scanner and LVShV was determined. At autopsy, the left ventricle was dissected and weighted. The correlation between LVShV and the anatomic LVM was analysed. Asymmetric left ventricular (LV) hypertrophy was recorded. Inter-observer variability was evaluated, and a density value for myocardial tissue was calculated.

**Results:**

The mean age of the deceased was 55 ± 16 years, and 58% was men. We found 30 cases of asymmetric LV hypertrophy. A highly positive correlation existed between LVShV and anatomic LVM (r = 0.857; *p* < 0.0001), regardless of hypertrophy, asymmetric hypertrophy and gender. The mean difference in the inter-observer variability for LVShV assessment was - 4.4 ml (95% CI: -26.4; 17.6). A linear regression analysis was performed, resulting in a value of 1.265 g/ml for myocardial tissue density. Applying the hitherto used myocardial tissue density of 1.055 g/ml underestimated the anatomic LVM by 18.1% (*p* < 0.0001).

**Conclusion:**

PMCT is a helpful tool for the assessment of LVM, and LVShV is highly correlated with LVM as assessed by subsequent autopsy. The correlation between the two was independent of gender, hypertrophy and LV asymmetric hypertrophy. We found a higher myocardial tissue density of 1.265 g/ml compared to previous studies. We show that PMCT combined with autopsy may contribute not only to anatomical but also clinical knowledge.

## Background

Left ventricular mass (LVM) is a prognostic factor in cardiac disease [1], and thus, assessment of LVM is used in the diagnosis and risk stratification of patients in clinical practice [2]. In addition, abnormal patterns of left ventricular (LV) size and shape have been found to have prognostic relevance [3–5]. Hypertrophy may occur in a specific region of the LV, asymmetric hypertrophy, regardless of the overall heart size [6, 7]. The most widespread tool for non-invasive measurements of the LVM is echocardiography [4, 7]. LVM determination by different imaging modalities is based on the LV shell volume (LVShV), which is the difference between the epicardial and endocardial volumes [4, 8]. The LVShV is subsequently converted to mass by multiplying it with the density of myocardial tissue [8, 9]. The clinically accepted value of myocardial tissue density is 1.055 g/ml [9–14]; however, there are different published values [6, 8, 15–22], cf. Table 1. LVM has been calculated based on Computed Tomography (CT)-angiography [10], magnetic resonance imaging (MRI) [23] and echocardiography [24], but the myocardial density has not been validated. The aim of this study was to assess the association between the LVShV determined by postmortem Computed Tomography (PMCT) and the anatomic LVM obtained at autopsy, and to calculate the myocardial tissue density**.**

**Table 1:** Previous studies investigating the myocardial tissue density or correlation between an image modality and the LVM

Studies describing the myocardial tissue density or myocardial tissue gravity						
Reference	Year	Material	Method	Number included	Density/gravity	Correlation, r
Friedmann C [21]	1951	Cadaver human hearts	Submersion in water	45	1.029	
Masshoff W [14]	1967	Cadaver human hearts	Submersion in water	23	1.055	
Webb A [35]	1979	Equine hearts	Archimedes principle	18	1.033	
Schapira JN [36]	1981	Canine hearts	Phased array sector scan	15	1.04	
Rufeng B [20]	2007	Cadaver human hearts	Density Instrument	169	1.3, 0.9	
Studies referring to a myocardial tissue density factor based on previous studies						
Snyder [18]	1975	Human hearts	Radiology	–	1.033	
Wyatt A [31]	1979	Canine hearts	Echocardiography	21	1.05	0.94, 0.92
Reicheck [17]	1981	Cadaver human hearts	Echocardiography, ekg	34	1.04	
Schiller [29]	1983	Canine	Echocardiography	10	1.055	0.98
Deveraux [12]	1986	Cadaver human hearts	Echocardiography	55	1.04	0.92
Manning J [30]	1990	Rat hearts	MRI	28	1.055	0.98
Jackowski C [13]	2004	Cadaver	MRI, CT-angio	80	1.05	
Lang [8]	2005	Living	Echocardiography	85	1.04	
Fuchs A [10]	2016	Living	CT-angiography	569	1.055	

## Methods

### Study population and design

The study population is part of a prospective nationwide autopsy-based Danish forensic study, **SURVIVE-let the dead help the living**, which focuses on deceased with mental illness [25]. The deceased were autopsied at the Section of Forensic Pathology at the Institute of Forensic Medicine, University of Copenhagen over an eleven-month period, from January 2014 to November 2014. The study group comprised 116 deceased that fulfilled the following inclusion criteria: individuals with a known or a suspected mental illness, and the intake of antipsychotic, antidepressive or anti-anxiety medication or a suspicion thereof. The exclusion criteria were putrefaction, suspicion of a criminal act, inadequate CT image quality for the analysis and failure to scan due to severe obesity. This led to the exclusion of seven individuals. After a medical inquest, the deceased underwent a whole-body PMCT. The scans occurred within 24 h prior to autopsy. At the autopsy (see details below), the sex, age, weight and height of the deceased were recorded, as were the size, shape and macroscopic changes of the heart. The project was approved by the Danish National Committee on Health Research Ethics (1377517).

### Post mortem CT imaging protocol

Non-contrast PMCT imaging was performed using a 64-slice Multi Detector Computed Tomography scanner (MDCT) (Siemens Somatom definition syngo 2010A; Siemens medical solutions, Forchheim, Germany). The following scanning parameters were used: tube current time: 300 mAs, tube voltage: 120 kV, slice collimation: 32 × 0.6 mm and slice acquisition: 64 × 0.6 mm. The iterative reconstructed slice thickness was 1.5 mm using a soft tissue convolution kernel. All images were acquired with the deceased wrapped in an artefact-free body bag in a supine position with elevated arms (except for one due to a BMI of 50). The bodies were placed in the CT gantry and scanned from head to toe. After reconstruction of the raw data, the images were transferred to the local PACS server.

### Imaging analysis

**Fig. 1:**
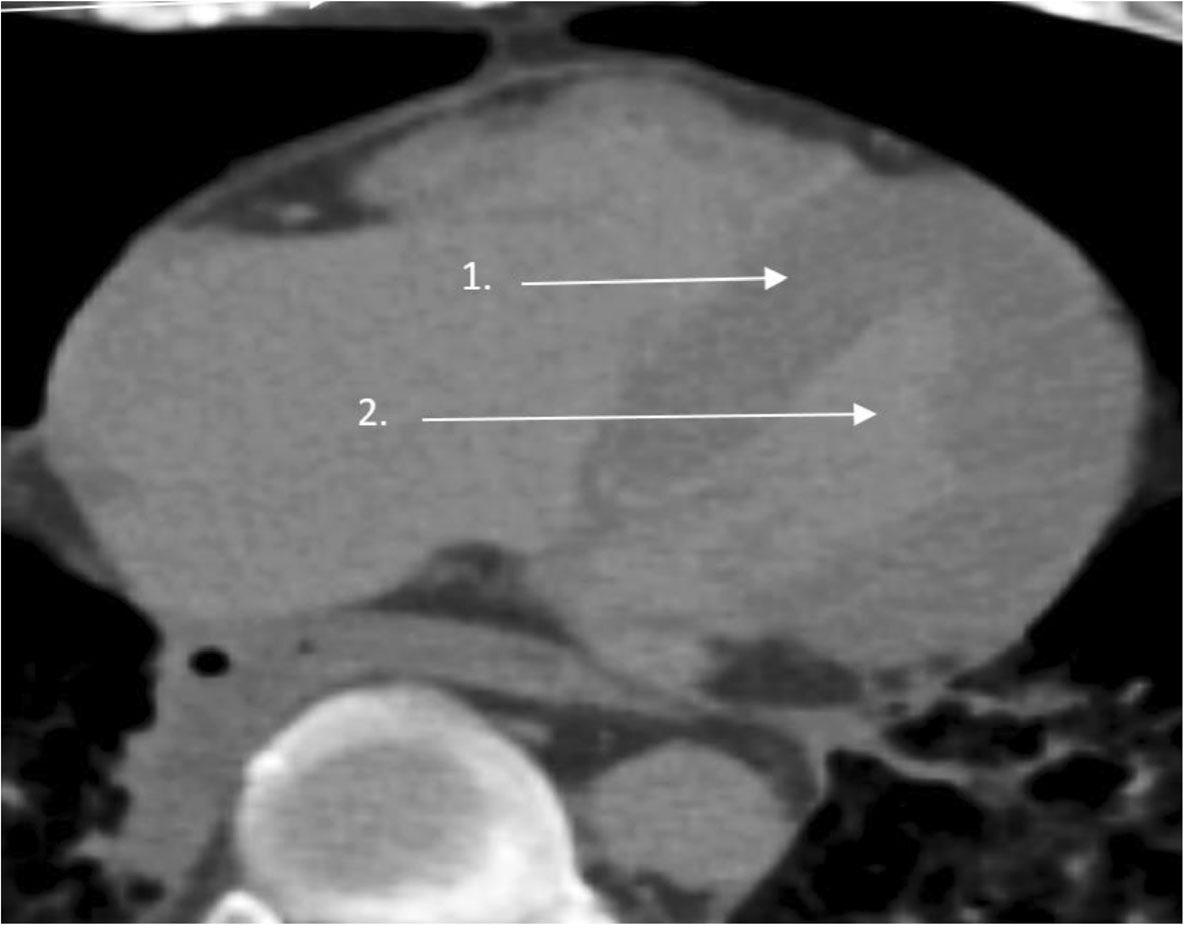
Long-axis wiev of the heart, illustrating the myocardial tissue (1) and the LV blood pool (2). The orange- and blue line indicates the epicardial and endocardial contours, respectively

Assessment of the myocardial tissue was performed with commercial software (Vitrea 6.3, Vital Images, Inc., MN, USA). Imaging analysis was performed by individuals who were blinded to the autopsy results. The non-contrast PMCT data were reviewed in multiplanar reconstructions after adjusting the planes to a long-axis view of the heart. The LV myocardial tissue was segmented by manually tracing the endocardial and epicardial border for each long-axis slice and adding up the corresponding volumes from each slice (Fig. 1). We used normal cardiac window settings with a level = 200 Hounsfield Units (HUs) and width = 1000. Voxels corresponding to myocardial tissue were identified using mean threshold attenuation values of 50 ± 10 HUs to distinguish it from the LV blood pool (Fig. 1). The region of interest (ROI) for the segmentation of both LV myocardial tissue and the LV blood pool was defined at the height of the septum and the LV outlet by HUs. The ROI was set at a minimum of 1 cm^2^. Depending on the heart size, the number of manually traced slices varied from 70 to 100 in each case. Finally, based on the segmentation, the LVShV was calculated in millilitres and converted to mass, by multiplying it with 1.055 g/ml. Inter-observer variability was assessed in 25 randomly chosen individuals. Fifteen randomly chosen LV myocardial tissues were re-analysed using a different cardiac setting with level = 45 HU and width = 50 HU.

### LV autopsy procedure

The autopsies were performed in accordance with the extended autopsy protocol of the **SURVIVE** study and the departments guidelines and were accredited by the Danish accreditation fund (DANAK). The length of the corpses were measured in the supine position from the top of the head (vertex) to the heel using an inelastic measuring tape. The heart was removed during the autopsy by preserving an adequate extension of the base and pulmonary vessels and dissected according to international standards detailed by Basso et al. [26]. After the removal of clotted blood, the whole heart was weighed. Dissection of the left ventricle was performed according to the method described by Bove et al. [27] with modifications; an incision was made in the posterior part of the left atrium following the atrioventricular groove to remove the atria from the ventricles. The ventricles were cut into transverse slices (short-axis direction). The right ventricle was separated from the septum and the left ventricle. The trabeculae encrusted in the septum and papillary muscles were preserved, and the hearts valves were removed. The epicardial fat was resected. The LV slices were blotted dry and separately weighed using an electronic scale (Mettler Toledo, ICS425, Glostrup, Denmark) with a 1-g precision.

The heart was recorded as being hypertrophic according to international forensic pathology standards and charts [28]. Anterior, lateral, posterior and septal wall thicknesses were measured at the level of the midventricular transversal slice. Papillary muscles were excluded from this measurement. Asymmetric LV hypertrophy was defined as biggest heart wall (millimetres) > 1.3*thinnest heart wall (millimetres) [7].

### Statistical analyses

Data analysis was performed using the SPSS statistical software package (IBM Corp. IBM SPSS Statistics for Windows, Version 24, USA). Data was presented as the mean ± standard deviation, and a *p-*value of < 0.05 was considered statistically significant. Data distribution was tested with the Ancova/ linear regression test. Bland-Altman analysis was used to assess the re-analysis of LVShV and the degree of inter-observer variability. Scatterplots were used to illustrate the level of agreement and linearity between the CT obtained LVShV and the anatomic LVM as well as asymmetric LV hypertrophy. The degree of correlation was determined by Pearson’s correlation coefficient. The association between the CT-obtained LVShV and the anatomic LVM, with and without adjustment for gender, was assessed by linear regression analysis. A density factor was calculated by linear regression analyses, forcing the slope through origo (as done in comparable studies) [7, 8].

## Results

Data were collected from a total study population of 109 deceased, comprising 46 females (42%) and 63 males (58%). The age and body weight distributions are detailed in Table 2. The deceased were autopsied at a mean time (± 1 SD) of 44 ± 16 h (range 29–150 h) after the declaration of death.

**Table 2:** Study group data, autopsy measurements and LVShV obtained by CT

	Women			Men		
	Mean	1 SD	Range	Mean	1 SD	Range
Age	59	18	21–94	52	14	22–79
Body weight, kg	67	67.4	38–130	79	15	54–120
Anatomic LVM, g	163	35	96–277	217	50	140–326
Anterior wall thickness, mm	13	3	9–18	13	3	8–20
Lateral wall thickness, mm	13	4	8–20	14	3	9–20
Posterior wall thickness, mm	13	4	9–25	13	3	9–18
Septal thickness, mm	14	3	9–20	14	4	8–21
Total heart weight, g	354	77	231–582	459	104	302–812
LVShV, ml	126.4	30.7	77–218	168.9	43.5	101–282

Descriptive data for study group and anatomic measures and CT determined LVShV (LVM, left ventricular mass; g, grams; mm, millimeters; kg, kilograms; LVShV, LV Shell volume)

### Left ventricular mass and LVShV

The results for anatomic LVM, LV wall thickness and LVShV are given in Table 2. There were 36 hypertrophic hearts, (25 men and 11 women) based on total heart weight. Thirty cases had asymmetric LV hypertrophy (19 men and 11 women) of which thirteen had hypertrophic hearts (total weight).

**Fig. 2:**
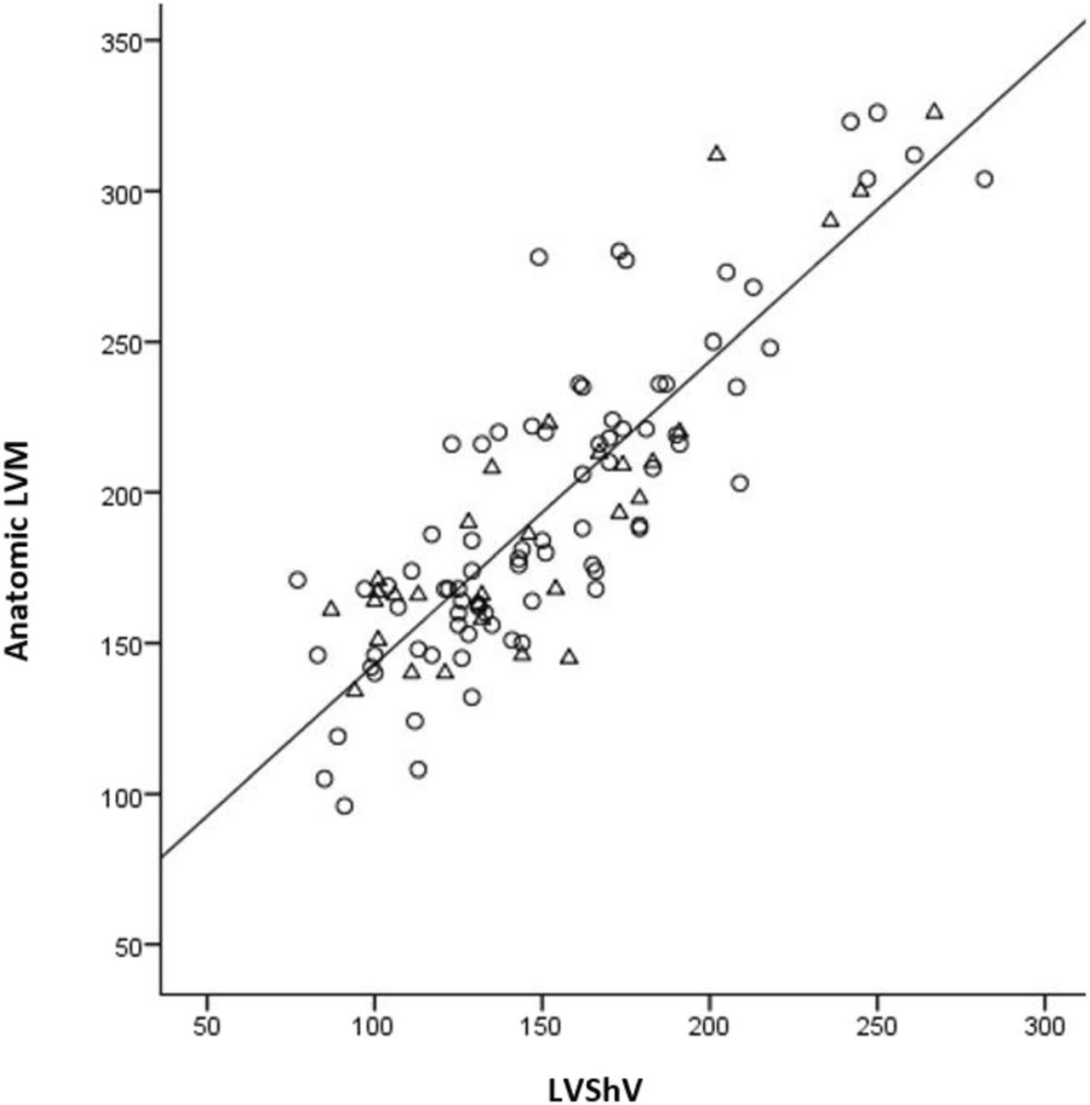
Correlation between the CT determined LVShV and anatomic LVM (Pearson r = 0.874, *p* < 0.0001) stratified by the presence of asymmetric LV hypertrophy (triangles)

**Fig. 3:**
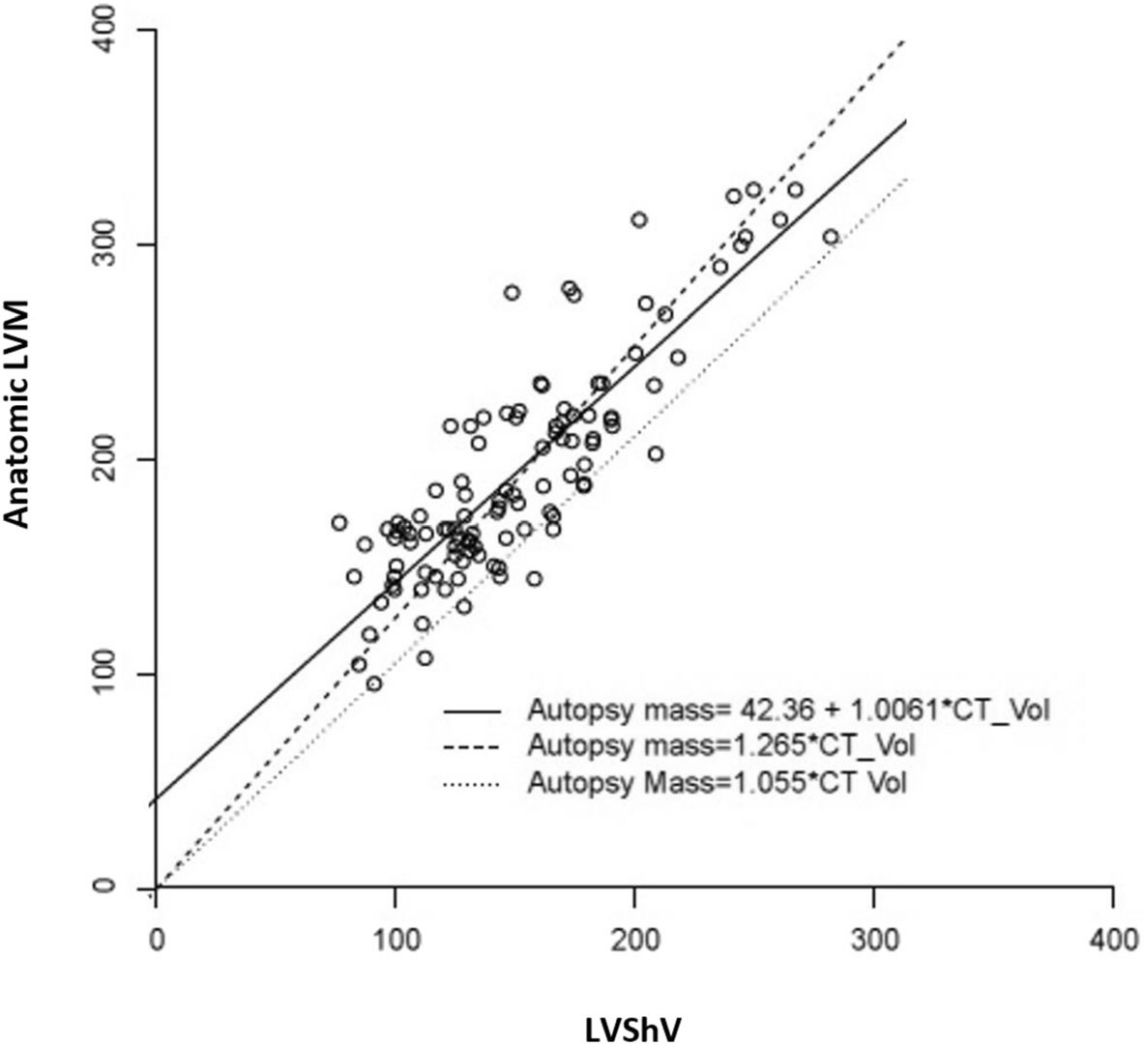
Regression equations lines for LVM by LVShV. The lines are for genders combined (with the constant of 42.36). CT_vol represents the CT determined LVShV and the autopsy mass represents the anatomic LVM. The plot also shows the underestimation of the anatomic LVM when applying the clinically used myocardial tissue density of 1.055g/ml

Overall, the CT-determined LVShV was highly correlated with the anatomic LVM (Pearson r = 0.857, *p* < 0.0001), also allowing us to assume linearity. Significant correlation was also found when the cohort was stratified by the presence of asymmetric LV hypertrophy (Fig. 2, Table 3) and by gender (Table 3). The inter-observer mean difference in LVShV assessment by CT was - 4.4 ml (95% CI: -26.4; 17.6). The mean HUs value of the ROI was 47 ± 7 in the segmentation of myocardial tissue and 68 ± 13 in the LV blood pool. A linear regression equation was calculated for each gender (Table 4) and for the genders combined (Fig. 3, Table 4). Linear regression analyses were also performed by forcing the line of regression through the origo for each gender (Table 4) and for the genders combined (Fig. 3, Table 4). The differences between the models based on gender were negligible. Forcing the line of regression through the origo resulted in the following estimated model: LVM = LVShV*1.265 g/ml, with 1.265 g/ml as the myocardial tissue density. The R^2^ values and residual standard error are presented in Table 4. Hypertrophic hearts based on total heart weight did not alter the myocardial tissue density compared to non-hypertrophic hearts (data not shown). Using the hitherto clinically accepted myocardial tissue density of 1.055 g/ml, the anatomic LVM was underestimated by 18.1% (95% CI = 29.9–40.0 g), *p* < 0.0001, (Fig. 3, Table 5). The mean difference in LVShV assessment by CT with the different settings in 15 randomly chosen LV myocardial tissues was - 19.4 ml (95% CI: -65.3; 25.5), *p* > 0.5.

**Table 3:** Correlation between the CT determined LVShV and anatomic LVM

	n	*R* value	*P* value
Women	46	0.739	<0.0001
Men	63	0.836	<0.0001
All	109	0.857	<0.0001
Asymmetric LV hypertrophy	30	0.874	<0.0001

Correlation values by gender and asymmetric LV hypertrophy

**Table 4:** Linear regression for the LVM by LVShV

	Linear Regression Equation	*R* ^2^	Residual SE	Linear regression forced through origo Equation	*R* ^2^	Residual SE
Women	45.73 + 0.93057 g/ml * LVShV	0.9835	26.15	1.267 g/ml* LVShV	0.9792	29.25
Men	59.63 + 0.93057 g/ml * LVShV	0.9835	26.15	1.264 g/ml* LVShV	0.9792	29.25
All	42.36 + 1.0061 g/ml * LVShV	0.7331	26.73	1.265 g/ml* LVShV	0.9792	29.11

There was no significant difference between the slope of the genders, why a fitted model was used, 0.93057. A slightly different constant was seen due to the LVM being bigger in men. There was no difference in the LVM calculating equation (LVShV * myocardial density), when the line of regression was forced through the origo. Knowing the gender and not forcing the linear regression through the origo had the best fit. (LVM, left ventricular mass; g, grams; ml, milliliters; LVShV, LV shell volume)

**Table 5:** LVM calculated using different myocardial tissue density and the anatomic LVM

	Women			Men			All		
	Mean	1 SD	Range	Mean	1 SD	Range	Mean	1SD	Range
LVM, g (LVShV*1.055 g/ml)	133	32	81–230	178	46	107–298	159	46	81–298
LVM, g (LVShV*1.265 g/ml)	160	39	97–276	213	55	127–357	191	55	97–357
Anatomic LVM, g	163	35	96–277	217	50	140–326	194	51	96–326

Resultant LVM using the different equations, with comparison to the anatomic LVM (LVM, left ventricular mass; g, grams; ml, milliliters; LVShV, LV shell volume)

## Discussion

As expected, our data showed a highly positive correlation between the CT-determined LVShV and the anatomic LVM; also when stratifying by gender and when focusing on cases with hypertrophy and asymmetric LV hypertrophy. We then performed linear regression analyses with LVShV as the explanatory variable and LVM as the dependent variable. Stratification by gender showed no differences. Potentially, the resultant regression equation (genders combined) could thus be used for calculating LVM when LVShV has been determined. Traditionally, clinical volume-to-mass calculations use only a density factor, i.e. the volume is simply multiplied by a density factor [8, 9]. This is in line with a theoretical approach to a density factor: zero volume equals zero mass. Earlier attempts at establishing a myocardial density factor have therefore, more or less explicitly, assumed that a volume-to-mass regression must go through origo [7, 12, 24], hence resulting in a simple factor, and not in a regression equation with both a factor and a constant. We therefore performed new linear regression analyses, but this time forcing the line of regression through the origo. Again, differences by gender were negligible, thus allowing us to propose the resultant slope for the combined genders, 1.265 g/ml, as the myocardial tissue density. This density value is based on CT-determined LVShV and the actual weight of the anatomic LVM obtained at autopsy. When we compared this new myocardial tissue density value with the hitherto used value in the clinical setting of 1.055 g/ml (6–15), we found that the latter value significantly underestimated the anatomic LVM.

Indeed, the assumed density of myocardial tissue has varied over time, e.g., 1.029, 1.03, 1.04 and 1.055 g/ml, not least because of differing techniques in determining the density, e.g., by immersing cardiac muscle tissue or hearts in water (Archimedes principle) [14, 20, 21], different image modalities and different animal species [12, 17, 29–32] (cf. Table 1).

Echocardiography is the most widespread tool used for the quantification of LVM [7, 8], CT- angiography is often used for LVM calculations [10] and cardiac MRI is often considered the gold standard for LVM assessment [23, 33]. Although not often used, non-contrast CT can also be used for information in LV size in the clinical setting [34]. In this large study of recently deceased individuals, we showed that the use of non-contrast PMCT for the determination of LVShV is a useful tool and has a satisfactory repeatability due to the fact that a clear distinction between the HUs of myocardial tissue and LV blood pool without active circulation could be made. However, these CT settings were optimized for contrast-based studies. Changing the CT settings in 15 randomly chosen cases showed an overall, but negligible and non-significant (*p* > 0.5), decrease in LVShV (mean difference − 19.4 ml). We did find that the image quality improved in some cases using these different settings (*n* = 7/15), as a more clear-cut distinction between the blood-pool and the myocardium was achieved. However, in other cases a lot of noise was introduced, so we find that the majority of the scans were in fact better analysed with the original settings.

Several studies have presented normal reference values for LVM based on CT-angiography [10, 11, 15], echocardiography [8, 24] and MRI [23, 33] and thus it is reasonable to develop modality-specific reference values. However, regardless of the imaging modality used to obtain the LVShV, all shell volumes are converted to mass by multiplying it with the myocardial tissue density [8], with 1.055 g/ml being the most commonly used density. To our knowledge, this study is the first to calculate the human myocardial density factor using non-contrast CT based LVShV and LVM obtained at autopsy. This may explain why our value differs from other proposed values. Since our value is higher, this means that a given LVShV will result in a higher LVM, and will be most pronounced for higher and potentially pathological LVShV, which may be of interest for clinicians and warrants further studies.

Although it is currently not fully accepted clinically to use the LVM as a regular test for patients [3], the exact CT measurements and accurate values for myocardial tissue density can lead to important therapeutic opportunities concerning diagnosis, treatment and prognostics. If the hitherto used density value is substituted with our value, this could have implications for the reference values as it will move some patients to higher LVMs indicating abnormal LVM. If one continues with the hitherto used density value, there will be no problem in using the reference intervals, but to the best of our knowledge, this would mean that the recorded LVM is not the real LVM.

### Study limitations

The following study limitations need to be addressed. This study included 73 non-hypertrophic hearts, but in order to develop LVM reference values based on the density, a bigger study population would be preferable. We did not take into consideration the status of ischaemic heart disease, fat infiltration or LV fibrosis and how this potentially could affect the size and shape of the left ventricle. The papillary muscles were included in the LVShV measurements. However, the impact of the inclusion or exclusion of papillary muscles on the assessment of LV function is negligible [15]. The scans were non-contrast scans, which in a clinical setting makes the differentiation between the LV blood pool and the LV myocardium difficult. However, non-contrast scans are possible in deceased individuals (cf. Fig. 1). We chose to perform the present study without contrast to avoid extravasation of contrast media in the examined deceased individuals and thereby omit a possible weight effect on the myocardium.

A study by Bai R et al. [20] has suggested a tendency of lower myocardial tissue density associated with pathological changes as oedema, none of our included cases had underwent putrefaction, as these were excluded. Therefore, we do assume that post mortal changes did not have any impact on the myocardial tissue density calculation. In vivo, the myocardium consist of intra-myocardial blood-volume. Postmortem, this volume may change, e.g., postmortem extravasation or, conversely, fluid accumulation from leaking and decomposing endocardial structures. Such changes are small and usually only become pronounced with extended postmortem intervals [35]. The deceased individuals in this study were kept at reduced temperature and autopsied rather quickly after declaration of death. Morphological observations as well as quantitative results suggest that elements of the blood are resistant to autolytic effects [36]. Overall, this leads us to assume that no significant organ volumetric changes took place. Water displacement was not performed in this study. There are several factors to take into consideration when performing water displacement measurements/techniques on cadaver hearts. Although it works accurately with solid objects, biological tissue is by its nature permeable and may be compressed or distended, and it may be fixed or unfixed. Given that we wanted to investigate CT-derived volume measures, as this is what is used clinically, we hence chose to base our volumes on this method. Boundaries are sensitive to threshold and windowing. We used a HU threshold for myocardial tissue of 50 ± 10, and 1 mm slice thickness for CT evaluations. The CT settings used for this study are optimized for contrast-based studies, and a different cardiac window settings could result in different myocardial volume. Also, different thresholds may apply for epicardial and endocardial borders and partial volume effects may be important. Due to the relatively close HU values of the blood pool and the myocardium, the myocardium may have been overestimated in some cases and underestimated in other cases**.**

Finally, our calculation of a new value for myocardial density is made performing a linear regression on the LVM and LVShV values of our study population. Thus, theoretically, we cannot be sure that very small hearts or LV masses below 96 g will conform to the linear model. This can probably only be investigated by also applying our method to a subadult study population. However, we note that all other analyses on myocardial tissue density were also constrained or even more limited. We would also note that when comparing with the hitherto-used value for myocardial density the differences are most pronounced for bigger hearts, so even if our new value has not been tested on very small hearts, any differences might be assumed minor.

## Conclusion

The unique access to both PMCT scans and autopsy measurements allowed us to assess the correlation between LVShV and the anatomic LVM; the analysis was also possible based on gender and LV asymmetric hypertrophy. Our regression models determined only very small differences when stratified by gender and negligible differences when forcing the regression through the origo, which allowed us to determine a myocardial density at 1.265 g/ml. Applying the hitherto used myocardial tissue density value (1.055 g/ml) significantly underestimated the LVM. Our proposed new value is the result of post-mortem CT for volume determination, followed by post-mortem dissection for obtaining LV weight. We argue that this allows for a more precise determination of these two basic parameters, but obviously, we are aware that post-mortem anatomy may not be directly translational to clinical studies. Several LVM reference value tables have been produced using myocardial tissue density value of 1.055 g/ml, thus continuing to use this value when converting from LVShV to LVM will not have immediate clinical implications. However, we do think that our study calls for critical evaluation of especially high LVMs and how this value is obtained.

## Abbreviations

CT: Computed Tomography
DANAK: Danish Accreditation fund
HUs: Hounsfield Units
LV: Left Ventricular
LVM: Left Ventricular Mass
LVShV: Left ventricular shell volume
MDCT: Multi Detector Computer Tomography
MRI: Magnetic Resonance Imaging
PMCT: Post Mortem Computed Tomography
ROI: Region of Interest
